# Pembrolizumab-Induced Checkpoint Inhibitor-Associated Diabetes Mellitus in Triple-Negative Breast Cancer: A Case Report

**DOI:** 10.7759/cureus.113405

**Published:** 2026-07-26

**Authors:** Shawlin Jahan, Haseen Ishraque Paabon, Kabyar Cho

**Affiliations:** 1 Department of General Internal Medicine, Furness General Hospital, Barrow-in-Furness, GBR; 2 Department of Medicine, Furness General Hospital, Barrow-in-Furness, GBR; 3 Department of Psychiatry, Furness General Hospital, Barrow-in-Furness, GBR

**Keywords:** ici-induced dm: immune checkpoint inhibitors-induced diabetes mellitus, immune-related adverse event (irae), insulin-dependent diabetes mellitus, pd-1 inhibitor, pembrolizumab, triple-negative breast carcinoma

## Abstract

Immune checkpoint inhibitors (ICIs) have transformed the management of multiple malignancies but can lead to immune-related adverse events (irAEs) affecting endocrine organs. Diabetes mellitus secondary to ICIs is uncommon but potentially life-threatening. We report a case of new-onset insulin-dependent diabetes developing during pembrolizumab therapy in a patient treated for triple-negative breast cancer. The patient presented with severe hyperglycaemia and ketosis after several cycles of immunotherapy. Autoimmune diabetes antibodies were negative, and C-peptide levels indicated relative insulin deficiency. Glycaemic control remained challenging after discharge, with alternating hyperglycaemia and hypoglycaemia despite insulin therapy. This case highlights the variability in the presentation of checkpoint inhibitor-associated diabetes and the challenges in ongoing glycaemic management.

## Introduction

Immune checkpoint inhibitors (ICIs) have transformed the treatment of a wide range of malignancies by enhancing anti-tumour immune responses through inhibition of key immune regulatory pathways. Programmed death-1 (PD-1) is an inhibitory receptor expressed on activated T-lymphocytes that normally helps prevent excessive immune activation and limits damage to healthy tissues. Pembrolizumab, a monoclonal antibody targeting PD-1, blocks this inhibitory signal, allowing T-cells to mount a stronger anti-tumour response. This approach has demonstrated significant survival benefits across several malignancies and is increasingly used in the neoadjuvant and adjuvant treatment of triple-negative breast cancer (TNBC) [1]. However, enhanced immune activation may also lead to immune-related adverse events (irAEs), in which normal organs become unintended targets of the immune response.

Endocrine irAEs are among the most frequently reported toxicities associated with ICIs and include thyroid dysfunction, hypophysitis, adrenal insufficiency, and insulin-dependent diabetes mellitus [2,3]. Unlike many other immune-mediated toxicities, endocrine complications are often irreversible because of the permanent destruction of hormone-producing cells and therefore frequently require lifelong hormone replacement therapy.

Checkpoint inhibitor-associated diabetes mellitus (CIADM) is a rare but potentially life-threatening endocrine irAE, with a reported incidence of less than 1%. It occurs predominantly in patients receiving PD-1 or programmed death ligand-1 (PD-L1) inhibitors, either alone or in combination with other ICIs [1,2]. Although uncommon, CIADM is clinically important because it typically develops abruptly and frequently presents with severe hyperglycaemia or diabetic ketoacidosis (DKA), which has been reported in 40-76% of cases and often requires emergency hospital admission [3].

The underlying pathophysiology is thought to involve immune-mediated destruction of pancreatic β-cells following disruption of PD-1/PD-L1 signalling [4,5]. Several studies have also identified genetic susceptibility factors that may increase the risk of checkpoint inhibitor-associated diabetes. Diabetes-associated HLA class II haplotypes, particularly HLA-DR4 and HLA-DR3, are over-represented in affected patients, suggesting a genetic predisposition to immune-mediated β-cell injury. More recently, human genomic studies have identified a missense variant in the *NLRC5* gene (Pro191Leu), a regulator of major histocompatibility complex (MHC) class I expression, as a potential susceptibility marker for ICI-induced diabetes, although its clinical utility requires further validation. However, CIADM differs from classical type 1 diabetes mellitus (T1DM) in several important respects. The onset is usually rapid, often occurring after initiation of immunotherapy, pancreatic autoantibodies are absent in a substantial proportion of patients, and residual β-cell function may still be present at diagnosis [5,6]. These features suggest that CIADM represents a heterogeneous condition rather than a uniform disease process and may make diagnosis challenging, particularly in patients who present without ketoacidosis or with atypical biochemical findings.

The timing of CIADM is also highly variable, ranging from days after initiation of immunotherapy to several months after treatment commencement, making continued clinical vigilance essential throughout treatment [2,3]. As the use of pembrolizumab continues to expand in breast cancer and other malignancies, early recognition and prompt management of this rare complication are essential to prevent potentially life-threatening metabolic decompensation.

We present a case of pembrolizumab-associated diabetes in a patient receiving neoadjuvant treatment for TNBC who developed severe hyperglycaemia with ketosis after multiple cycles of immunotherapy. This case is notable because the patient presented without detectable pancreatic autoantibodies and demonstrated evidence of relative rather than complete insulin deficiency at diagnosis, highlighting the clinical heterogeneity of CIADM. Furthermore, the subsequent challenges in achieving stable glycaemic control underscore the importance of close multidisciplinary collaboration between oncology and endocrinology teams in the management of patients receiving ICIs.

## Case presentation

A 60-year-old female patient presented with blurred vision, polyuria, polydipsia, and generalised fatigue, accompanied by significant unintentional weight loss of approximately 10 kg over 16 months. Capillary blood glucose at presentation was markedly elevated at 50 mmol/L.

The patient reported that episodes of blurred vision had previously occurred transiently following immunotherapy infusions and usually resolved within two days; however, on this occasion, the symptoms persisted for several days before improving following initiation of insulin therapy.

There were no associated infective symptoms, including fever, cough, shortness of breath, abdominal pain, nausea, vomiting, or changes in bowel habits, at initial presentation. There was no history of chest pain, syncope, or focal neurological deficits. The patient additionally reported recent dysuria. Chronic symptoms included fatigue and peripheral neuropathy affecting the hands and feet, which had been previously documented and remained stable without progression.

Past medical history was significant for X-linked hypophosphataemia, tertiary hyperparathyroidism status post parathyroidectomy, osteoporosis with Looser zones involving both femoral lesser trochanters and osteoarthritic hip changes, primary hypothyroidism, asthma, hypertension, and migraine.

The oncological history included triple-negative right breast invasive ductal carcinoma treated with neoadjuvant chemotherapy in combination with pembrolizumab, followed by breast-conserving surgery with a complete pathological response, adjuvant radiotherapy, and continuation of pembrolizumab therapy. Adjuvant bisphosphonate treatment had not been commenced because of underlying hypophosphataemia. The patient had recently completed the 13th cycle of pembrolizumab prior to this presentation.

Her body mass index (BMI) on admission was 29.4 kg/m². There was no family history of diabetes mellitus. Her previous HbA1c measured in 2024 was 37 mmol/mol, and previous random blood glucose measurements were within the normal range (5.3 mmol/L on 18 December 2025 and 6.2 mmol/L on 18 September 2025).

Investigations

Initial biochemical investigations demonstrated severe hyperglycaemia with ketosis but without acidosis. Venous blood gas analysis showed a glucose level of 50 mmol/L, ketones 3.0 mmol/L, pH 7.48, and bicarbonate 31.3 mmol/L. HbA1c on admission was 76. Routine laboratory investigations are summarised in Table 1, including mild hyponatraemia (129 mmol/L) and elevated FT4 (26.5 pmol/L), whereas diabetes-specific investigations, including pancreatic autoantibodies and C-peptide measurements, are presented in Table 2.

**Table 1:** Routine laboratory investigations at presentation. The results demonstrate the patient's biochemical profile, including mild hyponatraemia, and elevated FT4, during the initial evaluation of checkpoint inhibitor-associated diabetes. eGFR: estimated glomerular filtration rate; TSH: thyroid stimulating hormone; CRP: C-reactive protein; ESR: erythrocyte sedimentation rate; ALT: alanine aminotransferase; ALP: alkaline phosphatase.

Test	Result	Units	Reference range
Hb	142	g/L	115–165
WBC	6.4	×10⁹/L	4.0–10.0
Platelets	253	×10⁹/L	150–400
Sodium	129	mmol/L	133–146
Potassium	4.5	mmol/L	3.5–5.3
Creatinine	84	µmol/L	49–90
eGFR	65	mL/min/1.73m²	>60
FT4	26.5	pmol/L	7.9–14.0
FT3	4.2	Not stated	Not provided
TSH	1.262	mU/L	0.570–3.600
Cortisol (afternoon)	958	nmol/L	<276
CRP	<5	mg/L	<5
ESR	4	mm/hr	0–21
Inorganic phosphate	0.76	mmol/L	0.80–1.50
Adjusted calcium	2.65	mmol/L	2.20–2.60
ALT	25	IU/L	0–35
ALP	100	IU/L	30–130
Bilirubin	11	µmol/L	0–21
Albumin	45	g/L	35–50

**Table 2:** Diabetes investigations. Laboratory investigations demonstrating negative diabetes-associated autoantibodies and preserved residual β-cell function, supporting the diagnosis of checkpoint inhibitor-associated diabetes. GAD: glutamic acid decarboxylase; UCPCR: C-peptide:creatinine ratio.

Test	Result	Units	Reference range/cut-off
Anti-GAD antibodies	Negative	U/mL	Negative <11; positive ≥11
IA-2 islet autoantibodies	Negative	U/mL	Negative <7.5; positive ≥7.5
ZnT8 islet autoantibodies	Negative		Negative
Urine creatinine	3.0	mmol/L	Not provided
Urine C-peptide	1.59	nmol/L	Not provided
UCPCR	0.53	nmol/mmol	<0.2 Severe insulin deficiency; 0.2–0.6 intermediate; >0.6 substantial endogenous insulin secretion
Blood C-peptide	266	pmol/L	Normal: 370–1470; suppressed: <94; indeterminate: 94–300

As shown in Table 2, autoimmune diabetes markers (GAD, IA-2, and ZnT8 antibodies) were negative. The UCPCR of 0.53 nmol/mmol indicated intermediate endogenous insulin secretion, while the blood C-peptide concentration of 266 pmol/L was below the normal reference range, consistent with reduced but preserved β-cell function. Taken together with the abrupt onset of severe hyperglycaemia following ICI therapy, these findings support a diagnosis of checkpoint inhibitor-associated diabetes mellitus, characterised by significant insulin deficiency with residual endogenous insulin production rather than complete β-cell failure.

Treatment

The patient was initially managed with a variable-rate intravenous insulin infusion (VRII). Following review by the diabetes specialist nurse (DSN) team, the patient was transitioned to subcutaneous biphasic insulin therapy with Humulin M3, prescribed as 22 units in the morning and 14 units in the evening. The patient received education regarding glucose monitoring and insulin dose adjustment and was advised to increase the preceding insulin doses by increments of 2 units according to glucose readings.

Post-discharge course

Following discharge, the insulin regimen was modified in the community by the DSN team to a basal-bolus regimen consisting of Abasaglar (insulin glargine) 22 units in the evening and NovoRapid (insulin aspart) 8 units with each meal.

At follow-up, the patient remained on a basal-bolus insulin regimen comprising Abasaglar and NovoRapid. She continues to be reviewed regularly by the DSN, with ongoing insulin dose optimisation according to glycaemic control. Pembrolizumab therapy has been completed, and she remains under oncology follow-up every three months. A repeat HbA1c measurement was not available at the time of writing.

In addition, the patient developed diarrhoea, which began during the hospital admission and fluctuated following discharge. A faecal immunochemical test (FIT) was positive, and the patient is currently awaiting colonoscopy. The gastrointestinal symptoms raised concern for possible immune-related gastrointestinal toxicity associated with ICI therapy, although alternative gastrointestinal pathology remains under investigation.

## Discussion

ICIs are increasingly used as systemic cancer therapy today. In comparison to other cancer treatments, they work by reducing a person’s immune modulation and targeting the immune response against tumour tissue. There are a range of immunotherapy drugs available, including ipilimumab and tremelimumab (anti-CTLA-4), nivolumab and pembrolizumab (anti-PD-1), and avelumab, durvalumab, and atezolizumab (anti-PD-L1) [1].

Pembrolizumab is an IgG4 monoclonal antibody that is increasingly used in the treatment of multiple malignancies such as melanoma, breast cancer, non-small cell lung carcinoma, Hodgkin’s lymphoma, B-cell lymphomas, and urothelial carcinomas. Among irAEs, hyperglycaemia is a recognised complication [2]. However, ICI-associated diabetes is a rare irAE, occurring in fewer than 1% of patients. It is more commonly observed in patients receiving agents that target the PD-1/PD-L1 pathway, either as monotherapy or in combination with other ICI agents [3].

Clinically, CIADM typically presents with a rapid and fulminant onset of symptomatic hyperglycaemia, characterised by polyuria, polydipsia, weight loss, and often DKA. In a large case series, up to 60-76% of patients presented with DKA at diagnosis, highlighting the potentially life-threatening nature of this complication [4].

Unlike classical T1DM, many patients with CIADM lack detectable islet autoantibodies. In a very large retrospective cohort study analysing 14,000 patients, they identified three subtypes of ICI-induced diabetes based on pancreatic β-cell function and autoantibody status: (1) β+ patients with retained endogenous insulin production, (2) Ab+β− with absent β-cell function and positive islet-specific autoantibodies, and (3) Ab−β− with absent β-cell function and no detected islet-specific autoantibodies, with 88% of their cohort exhibiting loss of β-cell function [5]. They also found that patients with positive antibodies had a more rapid and acute presentation than patients with negative antibody status who presented in a more delayed onset. 

In systematic reviews, only approximately one-third of patients tested positive for antibodies such as GAD or IA-2, supporting a different immunological mechanism compared with classical autoimmune diabetes [4,6]. Another distinguishing feature is the rapid decline in C-peptide levels, reflecting acute β-cell destruction. Studies have demonstrated that many patients have markedly reduced or undetectable C-peptide at presentation or shortly thereafter [6,7].

Our patient received pembrolizumab for 13 cycles and developed the rapid onset of significant hyperglycaemia of 50 mmol/L, alongside symptoms of blurred vision, polyuria, polydipsia, and weight loss. Her HbA1c level was 76 mmol/mol; however, the rapid development of significant hyperglycaemia over eight days (with presentation occurring on day 8 after the 13th cycle) was atypical for the usual presentation of T2DM. She had a negative autoantibody screen (Table 2), which did not support classical T1DM or latent autoimmune diabetes of adulthood. Furthermore, the reduced but detectable C-peptide level shown in Table 2 suggested residual β-cell function rather than complete β-cell destruction.

This case highlights several features typical of CIADM, including abrupt onset of severe hyperglycaemia during pembrolizumab therapy and the absence of detectable diabetes autoantibodies. The pathophysiology of ICI-induced diabetes is not well understood. One proposed mechanism is immune-mediated destruction of pancreatic β-cells, resulting in insufficient insulin production, which closely resembles T1DM [4,6].

PD-1/PD-L1 inhibition may trigger pancreatic β-cell destruction through activation of autoreactive T cells. Activated autoreactive T cells release interferons (IFNs), which promote macrophage-mediated pancreatic β-cell destruction and progressive loss of endogenous insulin secretion [8]. PD-1 blockade by pembrolizumab results in immune hyperactivation through reactivation of autoreactive CD8+ T lymphocytes, leading to cytotoxic destruction of pancreatic β-cells and irreversible insulin deficiency.

In our case, the patient developed symptomatic severe hyperglycaemia at day 8 following the 13th cycle of pembrolizumab. Several reports have demonstrated that ICI-associated diabetes can present as early as five days post immunotherapy to several months or more than a year following discontinuation of the therapy, with rapid onset of DKA occurring in up to 40-76% of the patients. Most of these patients proceed to have irreversible insulin deficiency, necessitating lifelong insulin therapy [3]. A case series of nine individuals who presented with ICI-associated T1DM between 2015 and 2017 by Galligan et al. reported that the onset of diabetes was within the first month of commencing treatment in majority of these patients [9]. 

Currently, there are no blood tests or biomarkers available to screen for immunotherapy induced diabetes. The role of pancreatic autoantibodies remains unclear; there is variability in the current literature. Some studies have reported the presence of one or more positive autoantibody, primarily anti-GAD, in 30-50% of these patients who developed diabetes post immunotherapy; however, it is unclear whether these antibodies were present prior to the therapy or emerged as a result of seroconversion after exposure to ICIs [9]. Therefore, the prognostic value of autoantibodies in this setting appears limited. Routine blood glucose monitoring should be considered in patients receiving immunotherapy. 

Management generally requires lifelong insulin therapy, as recovery of pancreatic β-cell function is rare [10]. The necessity of long-term insulin therapy as observed in our patient, and reported in previous cases, supports the concept that immune-mediated β-cell destruction is largely irreversible. Multidisciplinary care involving oncology, endocrinology, and diabetes specialist nurses is recommended, particularly because patients receiving ICIs may simultaneously develop other endocrine irAEs.

## Conclusions

This case illustrates the development of checkpoint inhibitor-associated diabetes mellitus following pembrolizumab therapy for TNBC, presenting with severe hyperglycaemia and ketosis after multiple cycles of treatment. The diagnosis was supported by the abrupt onset of insulin-dependent diabetes in the setting of ICI therapy, despite negative pancreatic autoantibodies and evidence of preserved residual β-cell function, reflected by reduced but detectable C-peptide secretion. These findings highlight the clinical heterogeneity of checkpoint inhibitor-associated diabetes and demonstrate that complete β-cell failure may not be present at diagnosis.

This case emphasises the importance of maintaining a high index of suspicion for checkpoint inhibitor-associated diabetes in patients receiving PD-1 inhibitors who develop hyperglycaemia, even in the absence of classical autoimmune markers. Early recognition and prompt initiation of insulin therapy are essential to prevent life-threatening metabolic complications. Furthermore, close multidisciplinary collaboration between oncology, endocrinology, diabetes specialist nurses, and primary care teams is critical to optimise both oncological treatment and long-term glycaemic management. As the use of ICIs continues to expand, further studies are required to better define the clinical heterogeneity, underlying mechanisms, predictive biomarkers, and long-term outcomes of checkpoint inhibitor-associated diabetes.
